# The Role of the Niemann-Pick Disease, Type C1 Protein in Adipocyte Insulin Action

**DOI:** 10.1371/journal.pone.0095598

**Published:** 2014-04-21

**Authors:** Rachael Fletcher, Christopher Gribben, Xuiquan Ma, James G. Burchfield, Kristen C. Thomas, James R. Krycer, David E. James, Daniel J. Fazakerley

**Affiliations:** 1 Diabetes and Obesity Program, Garvan Institute of Medical Research, Sydney, New South Wales, Australia; 2 School of Biotechnology and Biomolecular Sciences, The University of New South Wales, Sydney, New South Wales, Australia; 3 Charles Perkins Centre, School of Molecular Bioscience, The University of Sydney, Sydney, Australia; Boston University School of Medicine, United States of America

## Abstract

The Niemann-Pick disease, type C1 (*NPC1)* gene encodes a transmembrane protein involved in cholesterol efflux from the lysosome. SNPs within *NPC1* have been associated with obesity and type 2 diabetes, and mice heterozygous or null for *NPC1* are insulin resistant. However, the molecular mechanism underpinning this association is currently undefined. This study aimed to investigate the effects of inhibiting NPC1 function on insulin action in adipocytes. Both pharmacological and genetic inhibition of NPC1 impaired insulin action. This impairment was evident at the level of insulin signalling and insulin-mediated glucose transport in the short term and decreased *GLUT4* expression due to reduced liver X receptor (LXR) transcriptional activity in the long-term. These data show that cholesterol homeostasis through NPC1 plays a crucial role in maintaining insulin action at multiple levels in adipocytes.

## Introduction

Metabolic diseases such as insulin resistance and type 2 diabetes (T2D) are major and escalating health concerns. Despite extensive research we have a rudimentary understanding of the molecular mechanisms that underpin insulin resistance in insulin target tissues. Recent advances in large-scale analysis platforms have offered the opportunity to reveal novel links between genes and disease phenotypes. For example, genome wide association studies (GWAS) have identified more than 70 genetic loci that associate with T2D [1]–[6], but for many of these the molecular basis for the association remains unknown. Studies to interrogate how genes associated with these loci impact whole body glucose metabolism are required.

One example of an associated gene is Niemann-Pick disease, type C1 (*NPC1)* which encodes a transmembrane protein that has been primarily studied in the context of Niemann-Pick type C (NPC) disease. NPC disease is a rare lysosomal storage disease characterized at the cellular level by the accumulation of cholesterol and sphingolipids within the late endosomal/lysosomal compartment [7]. NPC1 cooperates with NPC2 in the egress of cholesterol from the late endosomal/lysosomal system [8], [9], though the vast majority of NPC disease cases arise from mutations in *NPC1* [10]. Single nucleotide polymorphisms (SNPs) within the *NPC1* gene have been associated with early onset and morbid obesity and, independently of obesity, insulin resistance in humans [11]–[13]. Further, mice heterozygous for NPC1 have increased susceptibility to weight gain and display abnormal metabolic characteristics such as hyperinsulinemia and glucose intolerance [14], and this impairment in glucose tolerance is independent of body weight [15]. Taken together, these data suggest a role for NPC1 function in maintaining metabolic homeostasis and in predisposition to metabolic diseases.

NPC1 has been reported to influence insulin signalling in the spontaneous murine (nih) model of NPC disease [16]. Insulin acts largely via activating the serine/threonine Kinase Akt (PKB) through its phosphorylation at S473 and T308. NPC1 (nih) mice exhibited reduced phosphorylation of Akt and its substrate GSK3β in the brain, which was attributed to a marked decrease in insulin receptor substrate 1 (IRS1) expression. Also, pharmacological and genetic inhibition of NPC1 function reduced phosphorylation of Akt in endothelial cells due to inhibition of mTORC2 activity, which is responsible for phosphorylating Akt at S473 [17]. In light of the GWAS association, the metabolic profile of NPC1 null mice and these molecular data, we speculated that disruption of NPC1 function may impact metabolic homeostasis through reduced insulin-stimulated Akt activation and that the association between *NPC1* and insulin resistance may be due to effects on insulin action in its target tissues. We report that inhibition of NPC1 activity in an adipocyte cell model reduced insulin-stimulated glucose uptake through disruption of multiple sites: Akt activation, exocytosis of the insulin-responsive glucose transporter GLUT4, and total levels of GLUT4.

## Materials and Methods

### Cell Culture

3T3-L1 fibroblasts were cultured in Dulbecco’s modified Eagle’s medium (DMEM) (Life Technologies) supplemented with 10% foetal calf serum (Thermo Scientific) and 1% Glutamax (Life Technologies) at 37°C in 10% CO_2_. Fibroblasts were grown to confluence before differentiation into adipocytes in DMEM containing 100 ng/ml dexamethosone, 100 ng/ml biotin, 2 µg/ml insulin and 50 µM 3-isobutyl-1-methyl-xanthine (IBMX) for 3 days. Following this cells were post differentiated for a further 3 days in DMEM containing 2 µg/ml insulin. 3T3-L1 adipocytes were used for experiments 10–14 days after initiation of differentiation. HA-GLUT4 expressing 3T3-L1 adipocytes were generated as previously described [18]. Cells were treated for specified periods of time with 0.1% DMSO as a vehicle control or 10 µg/ml U18666a (Sigma-Aldrich). U18666a was not present during the basal period (DMEM, Glutamax, 0.2% BSA). Incubations with the LXR agonist GW3965 at 10 µM (Sigma-Aldrich) were performed during the final 16 h of 72 h U18666a treatment. Control (K1) and NPC1 knockout (2–2) CHO cells were a kind gift from Dr. Andrew Brown (UNSW, Sydney, Australia). CHO cells were cultured in DMEM F-12 nutrient medium supplemented with 10% foetal calf serum and 1% Glutamax at 37°C in 10% CO_2_.

### Transient Transfection and siRNA Mediated Knock Down

NPC1-YFP was kindly provided by Dr. Hongyuan Robert Yang (UNSW, Sydney, Australia). NPC1-YFP was transfected into CHO cells using Lipofectamine LTX (Life Technologies) following the manufacturer’s guidelines. For siRNA experiments approximately 2 million cells were used for each nucleofection with scrambed siRNA as a control (sense 5′-CAGTCGCGTTTGCGACTGGTT-3′) or siRNA to NPC1 (sense 5′-GGGAAAGAAUUCAUGAAAUUU-3′) (Thermo Scientific) using the Amaxa Kit and reagents following the manufacturers guidelines (Lonza). Cells were reseeded onto matrigel (BD Biosciences) coated plates and experiments were performed 4 d later.

### Preparation of Whole Cell Lysates and Western Blotting

Cells were serum starved in serum-free media for 2 h before incubation with 100 nM insulin for 20 min. Cells were lysed in lysis buffer (2% w/v SDS in phosphate buffered saline (PBS), pH 7.4, protease inhibitors (Roche), 1 mm sodium pyrophosphate, 2 mm sodium vanadate, 10 mm sodium fluoride) sonicated for 12s and centrifuged at 13,000×*g*. The protein concentration of the supernatant was determined by BCA assay (Thermo Scientific). 10 µg samples of protein were resolved by SDS-PAGE and transferred to PVDF membranes. Membranes were blocked in 5% skim milk powder in TBS-T (0.1% v/v Tween-20 in tris-buffered saline) for 1 h followed by an overnight incubation at 4°C with specific 1° antibody solutions. Membranes were incubated with an appropriate secondary antibody for 1 h before signals were detected using ECL (Thermo Scientific) or on the Odyssey infrared imaging system (LI-COR). Anti-Akt, anti-pT473 Akt and anti-pT308 Akt antibodies were obtained from Cell Signaling Technology. Anti-IRS1 antibody, anti-insulin receptor β antibody and anti-14-3-3 antibodies were from Santa Cruz Biotechnology. Anti-AS160 was from Upstate, anti-pT642 AS160 was from Symansis, anti-NPC1 antibody was from Abcam and anti-TfR antibody was from Zymed. IRAP, GLUT1 and GLUT4 antibodies were generated in-house.

### Filipin Staining

For imaging of U18666a-treated 3T3-L1 adipocytes cells were seeded into individual collagen type IV coated wells of µ-Slide 8 well chambered slides (Ibidi) prior to U18666a treatment as described above. Cells were at room temperature with 3% paraformaldehyde (PFA) in PBS for 30 minutes. PFA was quenched with 20 mM glycine and cells were blocked with 2% BSA in PBS. Filipin (Sigma-Aldrich) was added to cells at 125 µg/ml at room temperature for 30 min. Cells were imaged in PBS with 5% glycerol (Sigma) and 2.5% 1,4-diazabicyclo[2.2.2]octane (Sigma) on a Leica DMI 6000 SP8 MP microscope (Leica) using a 63×/1.20 water corrected objective. Z-stacks were acquired for the entire sample thickness up to 50 µm with a voxel size of 0.12×0.12×0.43 µm. The Filipin signal was imaged using multiphoton excitation at 725 nm and emission was detected at 450–550 nm.

For imaging of siRNA-treated cells, 3T3-L1 adipocytes were electroporated with scrambled siRNA or siRNA directed to NPC1 as described above. Cells were and seeded onto matrigel coated 42 mm coverslips (#1.5). Cells were fixed and stained as above. Cells were washed, mounted and imaged on a Leica DMI 6000 SP8 confocal microscope (Leica) using a 63×/1.20 water corrected objective. The Filipin signal was imaged using 405 nm excitation and emission was detected at 450–550 nm, voxel size 0.07×0.07×0.34 µm.

### Cholesterol Assay

3T3-L1 adipocytes were homogenised in HES buffer (10 mM Hepes, pH 7.4, 1 mM EDTA, 250 mM sucrose, protease inhibitors (Roche)) by 12 strokes of a dounce homogeniser. Cell debris was pelleted at 700×*g* for 10 min and a total membrane preparation acquired by centrifugation at 235,000×*g* for 75 min. Lipid was extracted by chloroform:methanol extraction. Briefly, membrane preparation was added to chloroform:methanol (2∶1) mix, vortexed and shaken for 10 minutes. ddH_2_O was added and samples were centrifuged for 10 minutes to generate a clear interface. The lower phase was removed and evaporated to dryness. Lipid residue was reconstituted in 100 µl 10% (v/v) Triton X-100 in Isopropanol and cholesterol levels determined using an Amplex Red based cholesterol detection assay following the manufacturer’s instructions (Life Technologies).

### 2-[3H]deoxyglucose Uptake Assay

3T3-L1 adipocytes were serum starved in serum-free DMEM for 2 hours before being washed in PBS (x3) and incubated in Krebs Ringer buffer (KRP) (0.6 mM Na_2_HPO_4_, 0.4 mM NaH_2_PO_4_, 120 mM NaCl, 6 mM KCl, 1 mM CaCl_2_, 1.2 mM MgSO_4_ and 12.5 mM Hepes (pH 7.4)) with 0.2% BSA. Cells were stimulated with 100 nM insulin for 20 min. To determine non-specific glucose uptake, 25 µM cytochalasin B was added into the wells before addition of 2-[3H]deoxyglucose. During the final 5 min, 2-deoxyglucose transport was initiated by addition of 2-[3H]deoxyglucose (PerkinElmer). Following three rapid washes in ice-cold PBS, cells were solubilized in 1% (v/v) Triton X-100 in PBS. Scintillation counts were normalized for protein content.

### Subcellular Fractionation

3T3-L1 adipocytes cultured in 15cm plates were harvested in HES buffer and dounce homogenised. Subcellular fractionation was carried out as previously described [19].

### HA-GLUT4 Assay

The determination of plasma membrane HA-GLUT4 levels was carried out as previously described [20].

### qPCR

3T3-L1 adipocytes cultured in 6 well plates were lysed in 1 mL TRIzol (Life Technologies). 0.2 mL of chloroform was added samples were mixed well. Samples were centrifuged at 12,000×*g* for 15 min at 4°C and the aqueous phase collected. Following addition of 0.5 mL 100% isopropanol, samples were incubated for 10 min at RT and centrifuged at 12,000×*g* for 10 min at 4°C. The pellet was washed with 1 mL 75% ethanol and centrifuged as 7,500×*g* for 5 min at 4°C. The RNA pellet was resuspended in RNase-free water and incubated at 60°C for 10–15 min. A master mix was prepared for the reverse-transcription reaction comprised of 10 x Buffer RT, 5 mM dNTP, 10 µM Oligo dT primer, 10 U/µl RNase inhibitor, Omniscript Reverse Transcriptase (Qiagen) and RNase-free water. 2 µg Template RNA was added to the master mix which was then briefly vortexed, centrifuged and incubated for 60 min at 37°C. qPCR was performed using the LightCycler 480 (Roche) using the LightCycler480 Master reagent (Roche), according to the manufacturer’s instructions. Primers for target genes were 5′-gcagatcaagcatcccaact-3′ and 5′- ccagagaatgtttcattgtcca-3′ for *ABCA1,* 5′- tgctctgggtaccatgacatc-3′ and 5′- cccacaaatgtcgcaacc -3′ for *ABCG1*, 5′-agaccctggaggctaaggac-3′ and 5′-agagccttcatcttcgcaat-3′ for *APOE, 5′-*catgagggaaatcaatgatcg-3′ and 5′-ctaaagctgagagagtgtgagagc-3′ for *TfR*, 5′-agtgttcatatctacgaaccgtacc-3′ and 5′-tcaatggcgatctgtaagtcc-3′ for *NPC1*, 5′-gacctgtcctcaagattggtg-3′ and 5′-gatggtgtgccttcatctgtc-3′ for *IRAP*, 5′- cagccggcacagctagag -3′ and 5′- agcactgctcctcccaca -3′ for *GLUT1*, 5′- gacggacactccatctgttg -3′ and 5′- gccacgatggagacatagc -3′ for *GLUT4* and 5′-ttcttcataaccacagtcaagacc-3′ and 5′-accttccgtaccacatccat-3′ for *CYPB*.

## Results

### Genetic Ablation of NPC1 Inhibited Insulin Signalling

**Figure 1:**
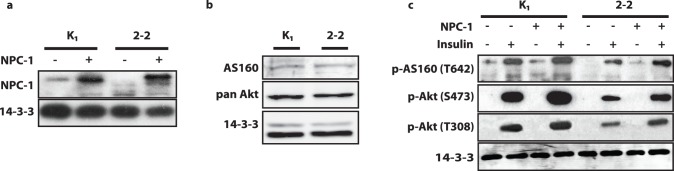
NPC1 knockout inhibited insulin signalling in CHO cells. (**a**) Total cell lysates of K1 CHO cells and 2–2 CHO cells were immunoblotted with anti-NPC1 antibody to determine expression levels of endogenous NPC1 and overexpressed NPC1-YFP. (**b**) Total cells lysates of K1 CHO cells and 2–2 CHO cells were immunoblotted with anti-Akt and AS160 antibodies to compare expression levels between K1 and 2–2 CHO cells. (**c**) Total cell lysates from unstimulated and insulin-stimulated cells (100 nM, 20 min) were immunoblotted with phospho-T308 Akt, phospho-S473 Akt, phospho-T642 AS160 and 14-3-3 antibodies to assess insulin signalling.

To initially assess the role of NPC1 activity on insulin signalling we utilised CHO 2–2 cells, an NPC1 knockout cell line [21]. These cells do not express NPC1 due to a frame-shift in the gene transcript (Figure 1A). We did not observe any changes in the total levels of the insulin signalling molecules Akt or Akt substrate AS160 (TBC1D4) between this cell line and wild type CHO (K1) cells (Figure 1B). However, differences were apparent upon insulin stimulation. In wild type CHO (K1) cells, insulin stimulated phosphorylation of Akt at S473 and T308 and AS160 at T642. This effect was reduced in 2–2 cells (Figure 1C).

We validated the specificity of this approach by rescuing NPC1 expression. NPC1-YFP was transfected into both K1 and in 2–2 cells (Figure 1A). NPC1 overexpression enhanced phosphorylation of Akt and AS160 in K1 cells suggesting that increasing NPC1 activity in wild type cells can augment insulin responses. Rescue of NPC1 expression in 2–2 cells restored insulin-stimulated phosphorylation of Akt at S473 and T308 and AS160 at T642 towards the levels observed in K1 cells, although the degree of phosphorylation at these sites did not fully revert to levels in control cells (Figure 1C). Overall, introduction of exogenous NPC1 improved signalling in both wild type and NPC1 KO cells.

### Inhibition of NPC1 Impaired Insulin Signalling in 3T3-L1 Adipocytes

**Figure 2:**
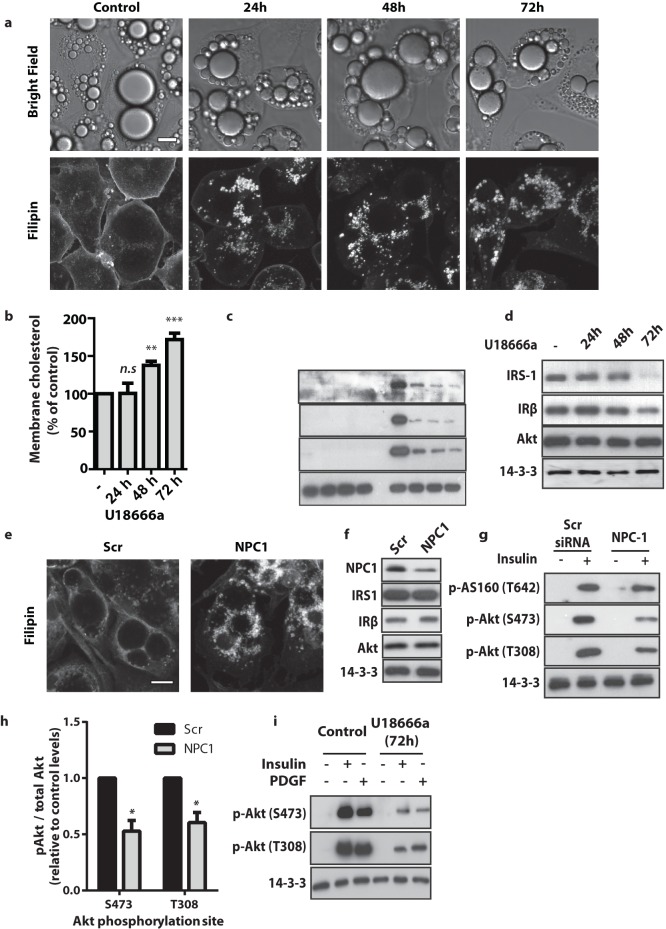
Pharmacological and genetic inhibition of NPC1 reduced insulin signalling in 3T3-L1 adipocytes. NPC1 was inhibited in 3T3-L1 adipocytes through treatment with U18666a or through siRNA-mediated knockdown of NPC1. (**a**) Subcellular localisation of cholesterol in 3T3-L1 adipocytes after treatment with U18666a for indicated times was visualised by Filipin staining (scale bar = 10 µm), bottom panel. Corresponding bright field images are provided (top panel). Images are representative of n ≥300 cells viewed per condition over 3 experiments. (**b**) Total membrane cholesterol levels within the total membrane fraction. Data are mean ± S.E.M, n = 3 independent experiments, one-way ANOVA. (**c**) Insulin signalling was assessed by immunoblotting total cell lysates of unstimulated and insulin-stimulated (100 nM, 20 min) 3T3-L1 adipocytes for phospho-T308 Akt, phospho-S473 Akt, phospho-T642 AS160 and 14-3-3. (**d**) Total protein expression of key proteins in the insulin pathway determined by immunoblotting for IRS-1, IRβ and Akt. (**e**) Subcellular localisation of cholesterol after treatment with scrambled or NPC1 siRNA was visualised by Filipin staining (scale bar = 10 µm). Images are representative of n≥300 cells viewed per condition over 3 experiments. (**f**) The extent of siRNA mediated knockdown of NPC1 and the effect of knockdown on expression of proximal insulin signalling components was measured by probing for NPC1, IRS-1, IRβ and Akt. (**g**) The effect of NPC1 knock-down on insulin signalling was assessed by immunoblotting with phospho-T308 Akt, phospho-S473 Akt, phospho-T642 AS160 and 14-3-3 antibodies. (**h**) The levels of insulin-stimulated pS473 and pT308 of Akt in cells NPC1 knockdown cells was quantified relative to control cells. Data are mean ± S.E.M, n = 3 independent experiments, two-sample t-test. (**i**) 3T3-L1 adipocytes overexpressing PDGF receptor were treated with U18666a for 72 hr prior to assessment of insulin and PDGF-mediated (20 ng/mL, 20 min) signalling. Signalling was assessed by immunoblotting with phospho-T308 Akt, phospho-S473 Akt and 14-3-3 antibodies. All data are representative of at least n = 3 independent experiments, significance calculated compared to control cells, n.s = non-significant, *, = p<0.05 ** = p<0.01 *** = p<0.001, statistical tests as indicated.

Having shown that NPC1 regulates insulin signalling in CHO cells, we investigated whether this link also exists in 3T3-L1 adipocytes, a more physiologically relevant cell model for insulin signalling. We first took a pharmacological approach to reduce NPC1 activity. U18666a is an agent used to induce an NPC disease phenotype and is reported to interact directly with NPC1 [22]. Incubation of adipocytes with U18666a for 24, 48, and 72 h resulted in redistribution of cholesterol from the plasma membrane to intracellular structures most likely due to the accumulation of cholesterol in lysosomes (Figure 2A). Treatment with U18666a also led to increased concentrations of cholesterol within the crude membrane fraction (Figure 2B). Both these readouts indicated effective NPC1 inhibition [7].

We used this model to interrogate the effect of NPC1 inhibition on insulin signalling in 3T3-L1 adipocytes. We treated 3T3-L1 adipocytes with U18666a for 24, 48, and 72 h prior to examining insulin signalling. Insulin-stimulated phosphorylation of Akt (T308 and S473), and AS160 (T642) was substantially reduced in U18666a treated cells (Figure 2C). To investigate why we observed this defect in insulin signalling, we measured the expression levels of proteins comprising key nodes within the insulin signalling pathway (Figure 2D). Total Akt levels were unchanged with U18666a treatment. However, expression of both the insulin receptor (IR) and insulin receptor substrate 1 (IRS1) were reduced in adipocytes incubated with U18666a for prolonged periods (48–72 h). However, these changes in total expression levels are unlikely to fully account for the reduced Akt phosphorylation since the change in IR and IRS1 occurred much later than the defect in Akt phosphorylation. In fact changes in IR and IRS1 corresponded better with redistribution of cholesterol from the PM to intracellular sites (Figure 2A).

### NPC1 Activity was Required for Full Akt Activation

To confirm our observations using a pharmacologic inhibitor of NPC1 we next used siRNA knock down to determine if this achieved the same outcome. Introduction of an NPC1-targetted siRNA into adipocytes was sufficient to achieve an approximately 50% reduction in cellular NPC1 levels and inhibit NPC1 function as indicated by the accumulation of cholesterol at intracellular sites (Figures 2E, F). Consistent with our studies with U18666a, phosphorylation of Akt at both 308 and 473 was reduced by approximately 50% (Figures 2G). In addition, AS160 phosphorylation at T642 was decreased in NPC1 knock down cells (Figures 2G). Intriguingly we did not observe any significant change in the levels of either IR or IRS1 with NPC1 siRNA (Figure 2F) in contrast to that seen with U18666a. This is consistent with our observation in drug treated cells the impairment in Akt signalling precedes the reduction in IR and IRS1 levels. To further confirm that defects at Akt were not due to reduced IRS levels we next examined the regulation of Akt by PDGF in 3T3-L1 adipocytes overexpressing the PDGF receptor. Unlike insulin, PGDF activates Akt through a direct interaction between the PDGF receptor and PI3K [23] and so initiation of Akt signalling in response to PDGF is independent of IRS1. We observed a reduction in PDGF-stimulated Akt activation in U18666a treated cells (Figure 2I). Based on these findings we conclude that that inhibition of NPC1 influences Akt function independently of its effects on IRS1 protein abundance.

### Inhibition of NPC1 Reduced Insulin-stimulated Glucose Uptake in 3T3-L1 Adipocytes

**Figure 3:**
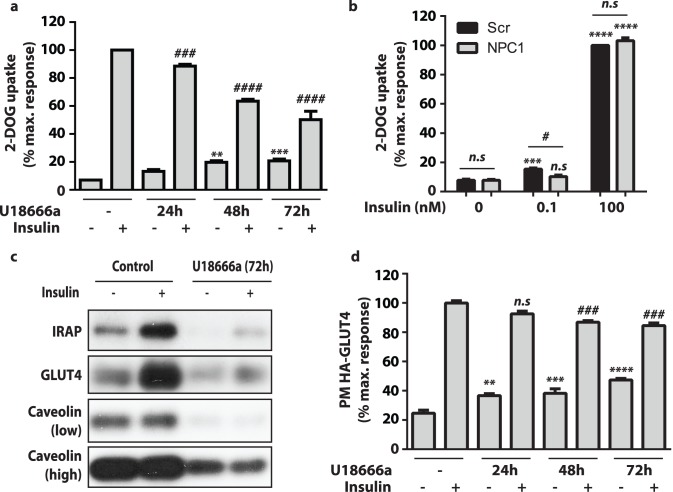
Inhibition of NPC1 repressed insulin-stimulated glucose uptake. (**a**) 3T3-L1 adipocytes were treated with U18666a for indicated periods of time before radiolabelled 2-deoxy-D-glucose uptake assays were performed on basal and insulin-stimulated cells. Data are mean ±S.E.M, n = 4 independent experiments. (**b**) 3T3-L1 adipocytes electroporated with scrambled (Scr) or siRNA directed to NPC1 (NPC1) were subjected to radiolabelled 2-deoxy-D-glucose uptake assays under basal and insulin-stimulated conditions. Insulin doses as indicated. Data are mean ±S.E.M, n = 3 independent experiments. (**c**) Plasma membrane (PM) levels of IRAP, GLUT4 and Caveolin 1 in unstimulated and insulin-stimulated (100 nM, 20 min) adipocytes were determined by immunoblotting. (**d**) PM levels of HA-GLUT4 was measured using a fluorescence based assay in 3T3-L1 adipocytes overexpressing HA-GLUT4 treated with U18666a for indicated periods of time. Data are mean ±S.E.M, n = 3 independent experiments. Significance calculated by two-way ANOVA. Comparisons to unstimulated cells (a, b, d) indicated by n.s = non-significant, * = p<0.05 ** = p<0.01 *** = p<0.001 **** = p<0.0001. Comparisons to insulin-stimulated cells (a, b, d) indicated by n.s = non-significant, # = p<0.05 ## = p<0.01 ### = p<0.001 #### = p<0.0001.

Since we observed considerable impairment of insulin signalling in response to NPC1 inhibition, we next measured whether this had downstream consequences; specifically on insulin-stimulated glucose transport. Insulin stimulates glucose transport in adipocytes through promoting trafficking of GLUT4 to the cell surface [24]. U18666a treatment substantially reduced insulin-stimulated 2-deoxyglucose uptake, and this phenotype worsened over the time course of NPC1 inhibition (Figure 3A). However, the extent of this defect was blunted compared to the level of Akt phosphorylation (Figure 2C). In addition, 2-deoxyglucose uptake in the absence of insulin was increased with U18666a treatment over time (Figure 3A). Although no defect in 2-deoxyglucose transport in NPC1-knockdown cells was observed at 100 nM insulin, there was a blunted effect at 0.1 nM (Figure 3B), indicating reduced sensitivity to insulin.

### Inhibition of NPC1 Altered GLUT4 Trafficking

Considering that basal and insulin-stimulated glucose uptake was affected by U18666a treatment, we hypothesised that GLUT4 trafficking to the PM in response to insulin would be altered in U18666a treated cells. We isolated PM fractions from unstimulated and insulin-stimulated adipocytes treated with or without U18666a and determined the levels of GLUT4 and IRAP, a protein that co-traffics with GLUT4 [25](Figure 3C). A striking observation was the reduction in PM GLUT4 and IRAP under basal conditions. PM caveolin levels were also reduced in U18666a treated cells. Although the major effect of U18666a treatment appeared to be in reducing GLUT4 levels in PM fractions from both basal and insulin-stimulated adipocytes, there also appeared to be a reduction in the fold-stimulation of GLUT4 translocation to the PM in U18666a-treated cells. To confirm this defect in GLUT4 translocation, we performed a complementary assay in 3T3-L1 adipocytes overexpressing GLUT4 with a HA-epitope in the primary exofacial loop (HA-GLUT4) to measure PM levels of GLUT4 [20]. The advantage of this assay is that PM HA-GLUT4 levels can be readily expressed relative to total HA-GLUT4 expression levels allowing determination of the level of HA-GLUT4 translocation to the PM independent of any effects on GLUT4 expression which may confound interpretation when assaying endogenous GLUT4 levels. PM GLUT4 was increased in the basal state and decreased in the insulin-stimulated state in response to U18666a. The extent of increased PM HA-GLUT4 under unstimulated conditions and inhibition of insulin-stimulated HA-GLUT4 translocation was more pronounced with increasing U18666a treatment length (Figure 3D). Overall, U18666a treatment resulted in modest but significant changes in GLUT4 trafficking under both unstimulated and insulin stimulated conditions.

### Total GLUT4 Levels were Reduced by U18666a Treatment

**Figure 4:**
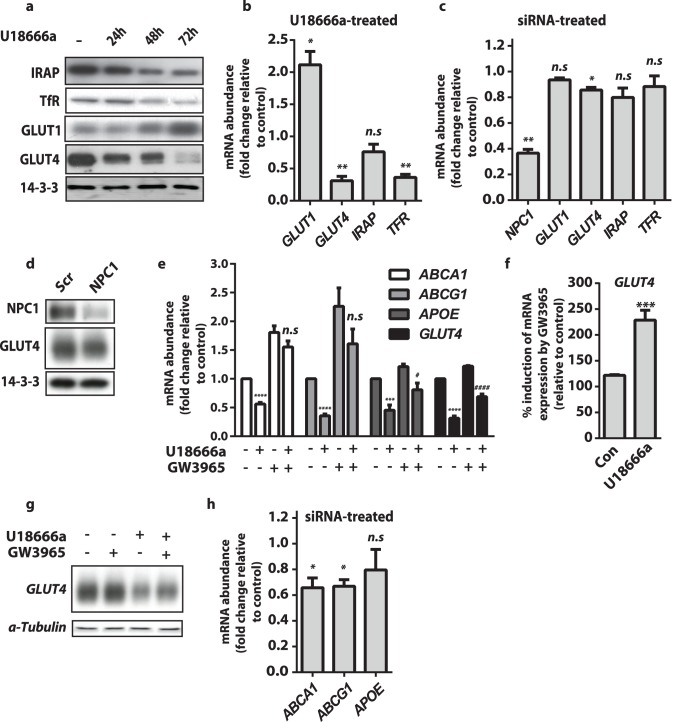
Inhibition of NPC1 reduced *GLUT4* expression via lower LXR activity. (**a**) Total protein levels of IRAP, Transferrin, GLUT1 and GLUT4 were determined by immunoblotting of whole cell lysates from cells with U18666a for indicated times. (**b**) *GLUT1, GLUT4, IRAP,* and *TFR* mRNA levels in 3T3-L1 adipocytes treated with U18666a for 72 h were determined by qPCR and presented relative to expression in control cells. Data are mean ±S.E.M, n = 3–5 independent experiments. (**c**) *NPC1, GLUT1, GLUT4, IRAP* and *TFR* mRNA levels in 3T3-L1 adipocytes treated with NPC1 siRNA were determined by qPCR and presented relative to expression in control cells. Data are mean ±S.E.M, n = 3 independent experiments. (**d**) Total protein levels of NPC1 and GLUT4 were determined by immunoblotting of whole cell lysates from control and NPC1 knockdown cells. (**e**) mRNA levels of *ABCA1*, *ABCG1* and *APO-E* and *GLUT4* were determined by qPCR. Control and U18666a (72 h) pre-treated adipocytes were treated with the LXR agonist GW3965 (10 µM) for 16 h. Data are mean ±S.E.M and expressed relative to mRNA levels for each gene in control cells. All data are n = 3–5 independent experiments**.** (**f**) Percentage change in *GLUT4* mRNA levels following GW3095 addition was calculated from (f). (**g**) Total GLUT4 protein levels were determined by immunoblotting of whole cell lysates from control cells and cells treated with U18666a and/or GW3095 as indicated. (**h**) *ABCA1, ABCG1 and APOE* mRNA levels in 3T3-L1 adipocytes treated with NPC1 siRNA were determined by qPCR and presented relative to expression in control cells. Data are mean ±S.E.M, n = 3 independent experiments. Significance calculated by two sample t-test (b,c,f,h) or two-way ANOVA (e) and indicated p values are compared to control cells n.s = non-significant, * = p<0.05 ** = p<0.01 *** = p<0.001 **** = p<0.0001 or cells treated with U18666a (f) n.s = non-significant, # = p<0.05 #### = p<0.0001 (e).

Despite observing changes in GLUT4 trafficking, the major observation from our subfractionation studies was reduced GLUT4 in the PM fraction. We next determined whether this was due to mislocalisation of GLUT4 or to a reduction in total levels of GLUT4. GLUT4 protein levels were considerably decreased, after 72h of U18666a treatment (Figure 4A). We compared this to effects on the levels of other trafficking proteins. Intriguingly the levels of IRAP and the transferrin receptor (TfR) were also reduced in cells exposed to U18666a for 72 h, but to a lesser extent than GLUT4. In contrast to effects on GLUT4, levels of another GLUT isoform, GLUT1, was increased in the U18666a treated cells (Figure 4A), indicating that U18666a treatment did not lead to a global reduction in trafficking protein expression.

### Inhibition of NPC1 Reduced LXR-mediated GLUT4 Transcription

To assess whether the change in GLUT isoform, IRAP and TfR protein expression was mediated transcriptionally, we measured transcript levels in control and U18666a treated cells by qPCR. U18666a resulted in down-regulation of *GLUT4* mRNA to 32% of that of control cells and of *TFR* to 36% of that in control cells (Figure 4B). In contrast, *GLUT1* transcription was up-regulated approximately 2-fold while *IRAP* transcript levels were unchanged implying that changes in IRAP protein were not mediated transcriptionally. We next measured mRNA levels of these genes in cells treated with NPC siRNA. This analysis confirmed NPC1 knockdown and also revealed a significant decrease in *GLUT4* expression which did not translate to a change in GLUT4 protein levels in these cells (89.9% ±7.1% S.E.M, n = 4, p = 0.25 by two sample t-test) (Figures 4C, D). The extent of *GLUT4* down-regulation at the mRNA and protein level was substantially less than observed in U18666a-treated cells. *GLUT1, TFR* and *IRAP* transcripts were not altered in NPC1 knockdown cells (Figure 4C).

It has previously been reported that the activity of the liver X receptor (LXR), a nuclear receptor that recognises oxysterol ligands, is reduced under NPC1 inhibition [26]. Further, LXR has been implicated in regulating *GLUT4* expression [27]. Therefore we tested whether LXR activity was reduced in adipocytes treated with U18666a through measuring the expression of classical LXR target genes *ABCA1, ABCG1*, and *APOE*. These genes were down-regulated by 44%, 64%, and 53% respectively, indicative of reduced LXR activity (Figure 4E). To test whether *GLUT4* expression was decreased as a function of reduced LXR activity we utilised GW3965, which activates LXR independently of cellular sterol status. GW3965 increased the expression of the classic LXR target genes as well as GLUT4 in control cells (Figure 4E). Applying GW3965 to cells pre-treated with U18666a for 72 h rescued the expression of *ABCA1* and *ABCG1* above the levels observed in untreated control cells, towards levels observed in control cells treated with GW3965. *APO-E* and *GLUT4* expression was increased towards levels observed in untreated control cells. For *GLUT4*, the increase in expression in response to GW3965 was significantly greater in cells pretreated with U18666a than in control cells (Figure 4E, F). This rescue of *GLUT4* mRNA levels also resulted in increased protein expression (Figure 4G). To ensure that reduced LXR activity was a result of NPC1 inhibition by U18666a and not an off target effect we also assessed LXR target gene expression in NPC1 knockdown cells. NPC1 knockdown altered LXR activity cells as *ABCA1* and *ABCG1* transcript levels were significantly reduced (Figure 4H). However, *APOE* levels were not changed. Nevertheless, these data indicate that decreased LXR activity partially accounts for reduced *GLUT4* gene expression upon NPC1 inhbition.

## Discussion

The molecular mechanisms of insulin resistance remain unresolved. Recent genome-wide studies have begun to reveal associations of genetic loci with insulin resistance. Indeed, GWAS data and genetic studies in mice have uncovered a link between insulin resistance and NPC1 [4]. Although this gene has been previously implicated in insulin resistance and glucose intolerance [14], the mechanism underpinning this association is undefined. Here we report that pharmacological or genetic inhibition of NPC1 function causes considerable defects at four major nodes of insulin signalling to glucose transport; at the level of IRS1 expression, Akt activation, GLUT4 trafficking and GLUT4 transcription. Additionally, these nodes appear to be differentially sensitive to NPC1 inhibition. These data strongly implicate NPC1 function and cholesterol homeostasis in the maintenance of insulin sensitivity in adipocytes.

One of the major phenotypes associated with reduced NPC1 levels or activity was reduced insulin signalling. Insulin-stimulated Akt and AS160 phosphorylation were reduced in an NPC1 KO CHO cell line (2–2) and in 3T3-L1 adipocytes in which NPC1 levels were depleted using siRNA (Figure 1C and 2G, H). The fact that this was observed in two cell lines supports the concept that NPC1 function is required in multiple cell types to maintain insulin responsiveness. U18666a treatments further disrupted insulin signalling to Akt and AS160 (Figure 2C), but also led to decreased levels of the insulin receptor and IRS1 (Figure 2D). This matches observations from NPC1−/− mice [16]. However, we did not observe the same reduction in these proximal signalling molecules in siRNA treated cells (Figure 2F). We speculate that this discrepancy may be due to the severity of NPC1 inhibition, with prolonged U18666a treatment possibly more potently inhibiting NPC1 function than the partial knockdown with siRNA. We observed a reduction in PM cholesterol and an increase in intracellular membrane cholesterol levels following U18666a treatment and siRNA-mediated NPC knock down (Figures 2A, B, E). However, only under conditions where PM cholesterol is undetectable relative to internal staining do we begin to observe reductions in IR and IRS1 levels (Figure 2A, D –48 h, 72 h). However, we cannot rule out that U18666a is having effects other than on NPC1, which may account for this difference. For example, U18666a is known to inhibit sterol synthesis [28], which could exacerbate the cholesterol trafficking defects observed in U18666a treated cells (Figure 2A). However, considering that cholesterol trafficking defects and IRS1 levels appear to correlate it may be that cholesterol-rich caveolae, within which IR resides [29], were disrupted by U18666a treatment. We observed a large reduction in caveolin at the PM in U18666a treated cells (Figure 3B) but no change in total cellular caveolin (data not shown), indicative of increased internal caveolin which has been reported to occur in response to PM cholesterol depletion [30]. Since there is evidence for a role for caveolin in stabilising IRS1 [31]–[33] and for caveolin as a promoter of insulin signalling [34], we favour the possibility that reduced IR and IRS1 levels are caused by a loss of PM cholesterol and subsequent loss of lipid rafts.

In addition to these IR and IRS1 effects, we also detected specific inhibition of Akt phosphorylation which was IRS1-independent. We provide several avenues of evidence for this conclusion. Firstly, we observed reduced Akt phosphorylation prior to impaired IRS1 expression in U18666a-treated cells, although we cannot rule out that IRS1 is mislocalised at earlier time points (Figure 2C and 2D). Secondly, a reduction in phospho-Akt was detected in siRNA-treated cells without changes in IR and IRS1 levels (Figures 2F, G). Thirdly, PDGF-dependent Akt signalling was also reduced (Figure 2I). These data indicate that NPC1 activity is required for complete activation of Akt and that this requirement is independent of the alterations in IRS1 protein levels. These findings shed new light on insulin signalling defects in the NPC1−/− mice, which had been previously attributed to solely reduced IRS1 expression [16]. Since we observed IRS1-independent inhibition of Akt phosphorylation in response to NPC1 inhibition, a defect is likely to lie at the level of phosphatidylinositol-3-kinase (PI3K) or at the upstream kinases of Akt, PDK1 and/or mTORC2. A number of components of this signalling nexus have been localised to cholesterol-rich lipid raft domains including phosphatidylinostol-2-phosphate (PIP2) [35]–[37] the substrate for PI3K and both PDK1 [38] and mTORC2 [39]. Further, PTEN, a negative regulator of Akt phosphorylation has been reported to be excluded from these domains [38]. Taken together, one possibility for the pronounced defect in insulin signalling downstream to Akt in the context of NPC1 inhibition is the loss of the localisation of these signalling molecules upon disruption of cholesterol trafficking.

Downstream of Akt signalling we also observed a defect in insulin-stimulated glucose transport following U18666a treatment (Figure 3A) and at low dose insulin in NPC1 knockdown cells (Figure 3B) indicative of NPC1 inhibition reducing insulin sensitivity. The time lag between reduced signalling and the defects in GLUT4 translocation and glucose uptake observed in U18666a-treated cells is of interest (Figures 2C, 3A,3D); U18666a treatment for 24 h caused a relatively small decrease in glucose transport and GLUT4 translocation, while Akt phosphorylation was considerably reduced at this time point. Furthermore, the level of insulin signalling is similar between 48 h and 72 h of U18666a treatment, whereas it is between these time points that major effects upon glucose uptake and GLUT4 translocation are observed. One explanation for this is the redundancy within the Akt signalling network. Only 5% of maximal Akt activation is required to elicit full glucose transport effects [40]. Additionally, disruption to other components comprising the insulin responsive system may also account for this. We observed increasingly aberrant GLUT4 trafficking over the 72 h U18666a treatment (Figures 2A, B). This manifested itself as increased PM GLUT4 (as a percentage of total levels) under unstimulated conditions and reduced insulin stimulated GLUT4 PM levels (Figure 3D). Decreased PM cholesterol has previously been reported to inhibit GLUT4 internalisation, which may explain increased proportion of total GLUT at the PM under basal conditions [41]. Further, disruption of cholesterol trafficking has been found to reduce SNARE machinery, specifically SNAP23 and syntaxin-4, at the PM [42]. These SNAREs mediate GLUT4 fusion with the PM [24] and thus impaired SNARE assembly at the PM or at other cellular organelles [43] may contribute to the reduced GLUT4 translocation in U18666a treated cells (Figure 3C and D).

Although we observed modest effects on glucose transport at times when signalling was reduced by U18666a (24 h) or siRNA-mediated NPC1 knockdown, the major disruption of regulated glucose transport only occurred in unison with large reductions in GLUT4 protein levels (table 1). Considering these data, we speculate that the metabolic complications associated with reduced NPC1 expression [14], [15] may be due, in part, to decreased GLUT4 expression in adipose since adipose-specific KO of GLUT4 confers insulin resistance and glucose intolerance [44]. Concomitant with decreases in *GLUT4* gene expression, we measured reduced in *TFR* but increased *GLUT1* expression (Figure 3B, C). Increased GLUT1 protein expression likely explains raised basal 2-deoxyglucose uptake rates observed in U18666a treated cells (Figure 3A). Although we detected a defect in GLUT4 trafficking in unstimulated cells under U18666a treatment (Figure 3D) this probably does not contribute to increased basal 2-deoxyglucose uptake as absolute levels of GLUT4 at the PM were severely reduced (Figure 3C).

**Table 1:** Summary of phenotypes with different modes of NPC1 inhibition.

	24 h U18666a	48 h U18666a	72 h U18666a	NPC1 siRNA
Akt signalling	↓↓	↓↓	↓↓	↓
GLUT4 translocation	=	↓↓	↓↓	NM
GLUT4 protein	↓	↓	↓↓	=
GLUT4 mRNA	NM	NM	↓↓	↓
**Glucose (2DOG) uptake**	↓↓	↓↓↓	↓↓↓	↓ (at low dose insulin)

Number of arrows indicates severity of phenotype. NM = not measured.

The effects of NPC1 inhibition described here are similar to those described in cholesterol depleted adipocytes [45]. This includes the change in expression of GLUT isoforms and induction of insulin resistance. Reduced cholesterol availability reduces LXR activity [46], which is a master transcriptional regulator of cholesterol homeostasis. LXR has been reported to regulate GLUT4 expression in adipocytes [27], and the LXR target gene *ABCA1* was reported to be down-regulated in cholesterol depleted adipocytes that also displayed reduced GLUT4 expression [45]. LXR activity was reduced in response to U18666a, as measured by target gene expression levels. The use of the LXR agonist GW3965 partially rescued the reduction in *GLUT4* gene expression (Figure 4F, G). In addition to *GLUT4*, *GLUT1* may also be LXR responsive. It has been shown previously to respond to an LXR agonist [47]; we found that GW3965 treatment similarly stimulated GLUT1 expression (data not shown). However, it responds differently to canonical LXR target genes in response to U18666a, suggesting that other transcriptional regulators are at play. Nevertheless, it is clear that changes in cholesterol homeostasis and LXR activity influence adipocyte GLUT isoform expression.

The data presented here indicate that it is the change in GLUT4 levels that has the biggest impact on insulin-regulated glucose transport and that it is possible to achieve marked reduction in Akt and AS160 phosphorylation without any major impact on insulin-regulated glucose transport at high doses of insulin. This is consistent with our previous work that highlights the spareness in signalling components comprising the PI3K/Akt signalling network [15], [48]. However, such spareness does not exist for GLUT4 and so models of GLUT4 repression are invariably associated with defective insulin action at the level of insulin-stimulated glucose transport. These include models of insulin resistance in 3T3-L1 adipocytes [49]–[51], transgenic models in mice [52], adipose tissue from insulin resistance mice [53] and adipose tissue from insulin resistant humans [54].

Inhibiting NPC1 and so disrupting cholesterol trafficking in 3T3-L1 adipocytes resulted in a complex phenotype culminating in a reduced response to insulin. Inhibiting NPC1 function resulted in both reductions in IR and IRS1 proteins levels possibly via instability created by disrupting cell surface lipid rafts and reduced activation of Akt by upstream kinases again possibly due to disruption of PM micro-domains. Further, it reduced GLUT4 levels partly through inhibition of LXR activity. Taken together, our data reveal the importance of NPC1 activity in the maintenance of adipocyte insulin sensitivity and implicates cholesterol homeostasis in regulating several nodes of the insulin response.
