# Correction: The role of the Niemann-Pick disease, type C1 protein in adipocyte insulin action

**DOI:** 10.1371/journal.pone.0103797

**Published:** 2014-07-22

**Authors:** The PLOS ONE Staff

In Figure 2C, the labels are missing. Please see the correct, complete Figure 2 here.

**Figure 2:**
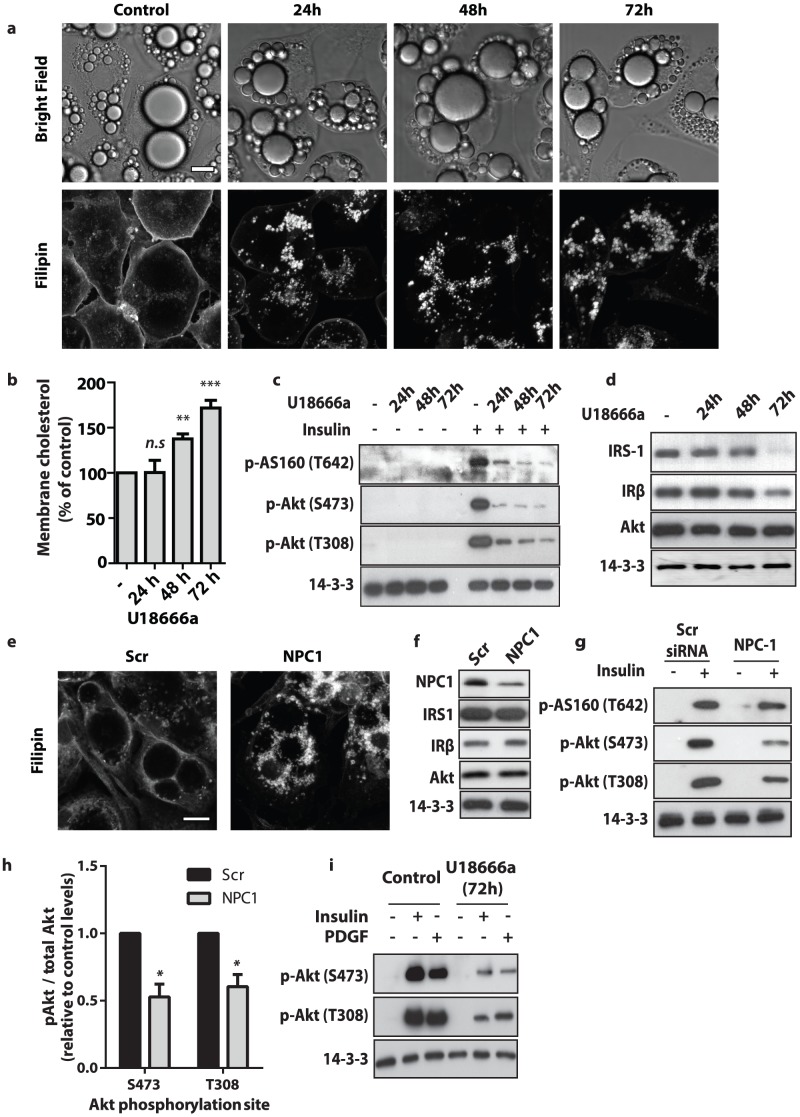
Pharmacological and genetic inhibition of NPC1 reduced insulin signalling in 3T3-L1 adipocytes. NPC1 was inhibited in 3T3-L1 adipocytes through treatment with U18666a or through siRNA-mediated knockdown of NPC1. (a) Subcellular localisation of cholesterol in 3T3-L1 adipocytes after treatment with U18666a for indicated times was visualised by Filipin staining (scale bar  =  10 µm), bottom panel. Corresponding bright field images are provided (top panel). Images are representative of n ≥300 cells viewed per condition over 3 experiments. (b) Total membrane cholesterol levels within the total membrane fraction. Data are mean ± S.E.M, n  =  3 independent experiments, one-way ANOVA. (c) Insulin signalling was assessed by immunoblotting total cell lysates of unstimulated and insulin-stimulated (100 nM, 20 min) 3T3-L1 adipocytes for phospho-T308 Akt, phospho-S473 Akt, phospho-T642 AS160 and 14-3-3. (d) Total protein expression of key proteins in the insulin pathway determined by immunoblotting for IRS-1, IRβ and Akt. (e) Subcellular localisation of cholesterol after treatment with scrambled or NPC1 siRNA was visualised by Filipin staining (scale bar  =  10 µm). Images are representative of n≥300 cells viewed per condition over 3 experiments. (f) The extent of siRNA mediated knockdown of NPC1 and the effect of knockdown on expression of proximal insulin signalling components was measured by probing for NPC1, IRS-1, IRβ and Akt. (g) The effect of NPC1 knock-down on insulin signalling was assessed by immunoblotting with phospho-T308 Akt, phospho-S473 Akt, phospho-T642 AS160 and 14-3-3 antibodies. (h) The levels of insulin-stimulated pS473 and pT308 of Akt in cells NPC1 knockdown cells was quantified relative to control cells. Data are mean ± S.E.M, n  =  3 independent experiments, two-sample t-test. (i) 3T3-L1 adipocytes overexpressing PDGF receptor were treated with U18666a for 72 hr prior to assessment of insulin and PDGF-mediated (20 ng/mL, 20 min) signalling. Signalling was assessed by immunoblotting with phospho-T308 Akt, phospho-S473 Akt and 14-3-3 antibodies. All data are representative of at least n  =  3 independent experiments, significance calculated compared to control cells, n.s  =  non-significant, *,  =  p<0.05 **  =  p<0.01 ***  =  p<0.001, statistical tests as indicated.
