# Correction: The Role of the Niemann-Pick Disease, Type C1 Protein in Adipocyte Insulin Action

**DOI:** 10.1371/journal.pone.0116042

**Published:** 2014-12-18

**Authors:** The PLOS ONE Staff

The third author's name is spelled incorrectly. The correct name is Xiuquan Ma.
